# Analysis of Lower Limb Asymmetry in Drop Jumps from Different Heights

**DOI:** 10.5114/jhk/194893

**Published:** 2025-05-29

**Authors:** I-Lin Wang, Chien-Ting Lai, Yu Su, Chin-Yi Gu

**Affiliations:** 1 College of Physical Education, Hubei Normal University, Huangshi, China.; 2 Graduate Institute of Sport Coaching Science, Chinese Culture University, Taipei City, Taiwan.; 3 Department of Rehabilitation Medicine, Hebei General Hospital, Shijiazhuang, Hebei, China.; 4 Department of Physical Education and Kinesiology, National Dong Hwa University, Shoufeng, Taiwan.

**Keywords:** impulse, shape factor, landing phase, side-to-side differences

## Abstract

This study aimed to investigate the effects of lower limb muscular asymmetry on the bilateral variations observed in high platform drop jumps, with a particular emphasis on the side-to-side differences in both the initial and secondary ground contacts during these jumps. Seventy-one students from the Physical Education department were selected to perform countermovement jumps. Participants were further classified into two groups, strength symmetry and strength asymmetry groups, based on the asymmetry strength index calculated from data collected via the force plates. Drop jumps were performed from three designated heights, i.e., 30 cm, 40 cm and 50 cm (DJ30, DJ40 and DJ50, respectively). The mixed design two-way ANOVA revealed notable differences between the stronger and weaker legs. Significant differences were observed in the timing of the peak vertical ground reaction force at the first ground contact of the DJ30 (p < 0.05) and in the shape factor of the push-off phase of the DJ40 (p < 0.05). The unilateral preference might lead to an increased risk of lower limb injuries. Prolonged training may exacerbate the degree of lower limb asymmetry. Potentially these findings can provide valuable suggestions for athletes and coaches in their training.

## Introduction

In most sports, actions such as jumping and landing are performed using both limbs. The difference in neuromuscular response between the dominant leg (DL) and the non-dominant leg (NDL) during dynamic lower limb jumping task landing is one of the main concerns of lower limb injuries (Sinsurin et al., 2017), and these muscles are considered to be the main auxiliary source of shock absorption during landing due to their ability to absorb kinetic energy. Thus, reinforcing muscle strength around the hip and knee joints of the lower limb could cushion the impact of the landing and prevent injuries (Zhang et al., 2008). The drop jump (DJ) is widely used in training as a plyometric exercise, and bilateral lower extremity differences are not only associated with landing injuries, but may also further affect athletic performance of individuals during secondary jumps (Gu et al., 2021; Wang et al., 2022). Therefore, bilateral asymmetry warrants further in-depth exploration in regard to its application in assessing lower extremity injury risk. During the instant of landing, the human body must bear the vertical ground reaction force (vGRF). After the landing from a vertical jump, the resulting vGRF is approximately 3.5 to 7.1 times the body weight (Gross and Nelson, 1988). When landing after a jump from a higher elevation, peak vGRF is generated, thus resulting in greater loads on the lower limbs (McNitt-Gray, 1991; Wang et al., 2015). The vGRF during the landing phase is an indicator of the landing load and the risk of injury.

When evaluating injuries and discrepancies in lower limb dynamics due to bilateral asymmetry, no significant difference was found between the DL and the NDL (Cadens et al., 2023; González-Ravé et al., 2014; McPherson et al., 2016; Skalski et al., 2024; Wang and Fu, 2019). Previous research has indicated that the DL is not always the stronger leg (SL), and sport-specific characteristics and training years often enhance strength of the more utilized leg (Dello Iacono et al., 2016; Menzel et al., 2013). If this difference in muscular strength is used as a basis for distinction, then there exists a disparity between the two sides (Impellizzeri et al., 2007; McLean and Tumilty, 1993). Advanced methods such as isokinetic testing or force plate measurements are essential to investigate disparities between the SL and the weaker leg (WL). In recent sports science research, several different terms have been used to examine muscle strength asymmetry: the bilateral strength asymmetry index (BAI), the limb symmetry index (LSI), the asymmetry index (ASI), and the symmetry index (SI). These indices are vital for assessing both performance differences and injury risk and serve as key evaluation metrics in studies on athletes' return to sports post injury (Lambert et al., 2020; Webster et al., 2019).

In the analysis of bilateral asymmetry, specific moments such as the timing and magnitude of the initial and peak vGRF are often selected as key variables. This approach is known as discrete point analysis (DPA) (Richter et al., 2014). However, human movement represents a continuous sequence over time, with the manifestation of bilateral asymmetry extending beyond isolated instances to encompass the entire movement process. This fact raises a critical question: does relying solely on DPA provide comprehensive and accurate representation of bilateral asymmetry? In response to this concern, there has been an increasing shift in focus toward examining the global characteristics of the movement curve. This holistic approach aims to capture the complete dynamics of motion. For instance, Peng et al. (2019) investigated the occurrence of multiple peak values within a curve, while Sole et al. (2018) analysed the shape factor of a curve. Moreover, Richter et al. (2014) expanded this inquiry through continuous data analysis. Collectively, these studies underscore the importance and relevance of evaluating overall curve characteristics to gain a deeper understanding of bilateral asymmetry in counter movement jumps (CMJs).

However, whether the bilateral differences in these movements are related to the asymmetry of strength in both limbs and whether they persist throughout the entire sports process must be clarified. Therefore, in this study we explored bilateral differences in landing movements through DJs from a high platform using the DL/NDL and the SL/WL as comparative distinctions. Moreover, bilateral differences were analysed, and curve morphology features were observed to gain a deeper understanding of the issues of lower limb strength asymmetry and bilateral differences in the landing. The hypothesis of this study was that there would be bilateral differences in timing, ground reaction forces, and shape factors during DJs of different heights between groups with symmetric muscle strength in both legs and groups with asymmetric muscle strength.

## Methods

### Participants

Using G*POWER 3.0 software (Universität Düsseldorf, Düsseldorf, Germany), analysis with a power value of 0.80, a Cohen’s function size of 0.3 and an alpha level of 0.05 determined that 26 individuals constituted the minimum number of study participants. Nevertheless, for this study, we initially recruited 71 male participants from sports-related departments who were in good health and had no history of lower limb surgeries or injuries in the six months prior to the study. Participants were first asked to perform a test of bilateral CMJs in the laboratory. After the test, the ASI of bilateral leg strength was calculated using the data from the force plates, *ASI* = [( *SL*− *WL*)/*SL*] × 100% (Fort-Vanmeerhaeghe et al., 2016, 2023; Newton et al., 2006). The average and standard deviation of the ASI were calculated for the 71 subjects (4.39 ± 3.18). Individuals with an ASI greater than the average ASI > ASI*Mean*+ 0.5 *SD* = 5.98 were assigned to the asymmetry strength group (AS group), while those with an ASI less than the average ASI < ASI*Mean* − 0.5*SD* were assigned to the symmetry strength group (SS group) (Peng et al., 2019). Individuals with an ASI that fell between these two groups were excluded. Those willing to participate in the second phase of the experiment were recruited from the remaining subjects. The study was approved by the National Taiwan Normal university ethics committee (protocol code: 201912EM0l9; approval date: 18 February 2020) and it was conducted following the principles of the Declaration of Helsinki.

### Procedures

The experiment was held at least four days after the CMJ test to avoid the impact of soreness. Before testing, participants filled out a consent form and were briefed for 20 min on the study purpose, process, and precautions, yet were not provided with the information regarding their group assignment to avoid bias (they were informed about it post experiment only). All participants wore the same model of shoes during the test to control for variance. After a 15-min warm-up (5-min treadmill jog at 7 km/h and 10 min of dynamic exercises), participants rested for 5 min. For the DJ (Gu et al., 2021; Heebner et al., 2017), participants stood on a platform with their hands on their hips, ensuring separate landing for each foot on different force plates (Gu et al., 2021; Wang et al., 2021). They fell forward, rebounded upon ground contact, aiming for a vertical jump, and then quickly attempted to jump up again as soon as possible after the first landing. The tester verbally reminded all participants to jump upwards as quickly as possible after touching the ground (Furuhashi et al., 2023; You and Huang, 2022). The DJ was tested at heights of 30 cm, 40 cm and 50 cm (DJ30, DJ40 and DJ50, respectively). Throughout, participants kept their hands on their hips, avoiding swinging. In this study, two AMTI (BP600900, AMTI, Inc., Watertown, MA, USA) three-axis force plates (2000 Hz) and eight optical cameras (200 Hz) were used to measure GRF variables and reflective ball trajectory data of participants via Qualisys Track Manager motion capture software (Innovative Sports Training, Inc., Chicago, IL, USA).

### Statistical Analysis

The SPSS (version 14.0; IBM Corp., Armonk, NY, USA) statistical package was used for statistical analysis. All results are presented as descriptive statistics with means and standard deviations. Independent sample *t*-tests were used to analyse the bilateral differences in various biomechanical variables during the landing phase of each platform between the SS and the AS group. When bilateral differences between two groups reached statistical significance, further two-way ANOVA (mixed design) was conducted to test whether there were differences in bilateral differences between the SS group and the AS group in the SL and the WL. If there was a significant interaction effect, a simple main effect analysis was conducted; if the interaction effect was not significant, a main effect analysis was performed. The level of statistical significance was set at *α* = 0.05.

## Results

### Lower Limb Strength Characteristics

In this study, through the first phase of countermovement jump tests, participants were divided into SS and AS groups, totaling 26 subjects (12 in the SS group and 14 in the AS group). In the SS group, three participants had their left leg as the DL and nine participants had the right leg as the DL, with eight participants having the DL and the SL on the same side. In the AS group, five participants had their left leg as the DL, and nine participants had their right leg as the DL, with 12 participants having the DL and the SL on the same side. Lower limb strength data obtained from the first-phase reverse jump test are shown in Table 1. The interaction effect between two factors (Group*Strength) was significant (*p* < 0.001). The simple main effects revealed that there was no significant difference between the groups; however, within the AS group, there was a significant difference between the SL and the WL (*p* < 0.001).

**Table 1:** The SS and AS groups’ strength in the SL and the WL at the first stage.

			*p* values		
	SL (BW)	WL (BW)	Main effect (Group)	Main effect (Strength)	Interaction (Group×Strength)
SS group	1.27 ± 0.15^a^	1.26 ± 0.16 ^b^	*p =* 0.110	*p <* 0.001*	*p* < 0.001*
AS group	1.36 ± 0.13 ^a^	1.24 ± 0.13 ^b^			

SL: stronger leg; WL: weaker leg; BW: body weight; SS: symmetry strength; AS: asymmetry strength; ^a^ indicates a significant difference compared to the WL, p < 0.05; ^b^ indicates a significant difference compared to the SL, p < 0.05

### Bilateral Differences in Landings from Different Heights

**Figure 1:**
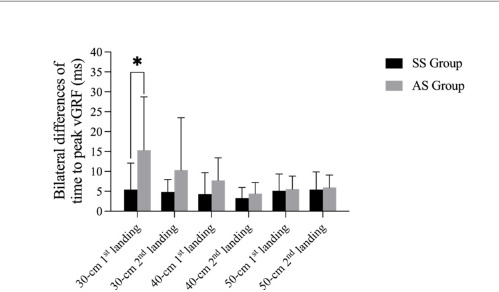
Bilateral differences in the 1^st^ and the 2^nd^ time to peak vGRF during drop jumps from different heights between SS and AS groups. * significant differences between the SS and the AS group

**Figure 2:**
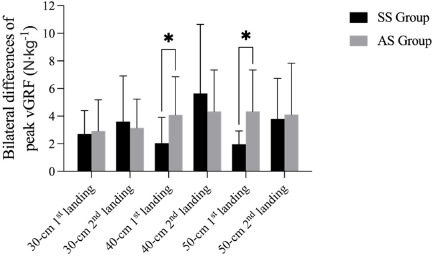
Bilateral differences in the 1^st^ and the 2^nd^ peak vGRF during drop jumps from different heights between SS and AS groups. * significant differences between the SS and the AS group

**Figure 3:**
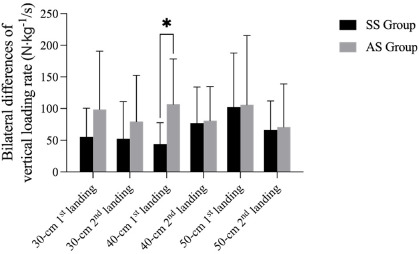
Bilateral differences in the 1^st^ and the 2^nd^ vertical loading rate during drop jumps from different heights between SS and AS groups. * significant differences between the SS and the AS group

Bilateral differences of time to peak vGRF, peak vGRF, and the vertical loading rate during the landing phase are shown in Figures 1– 3. The results revealed a significant difference of time to peak vGRF during the DJ30 between the SS and AS groups, with a significantly lower mean value (*p* = 0.029; 5.42 ± 6.67 ms; 15.36 ± 13.34 ms). Considering the DJ40, there was a significant difference (*p* < 0.05) in the first peak vGRF (*p* = 0.043; SS = 2.05 ± 1.86 N∙kg^−1^, AS = 4.09 ± 2.77 N∙kg^−1^) and in the vertical loading rate (*p* = 0.011; SS = 43.97 ± 33.68 N∙kg^−1^/s, AS = 106.84 ± 71.61 N∙kg^−1^/s) between the SS and AS groups. Finally, with regard to the DJ50, there was a significant difference (*p* < 0.05) in the peak vGRF between the SS and AS groups (*p* = 0.015; SS = 1.96 ± 0.97 N∙kg^−1^, AS = 4.35 ± 2.99 N∙kg^−1^). However, there were no significant differences (*p* > 0.05) of time to the first or the second peak vGRF, the values of the peak vGRF and the vertical loading rates.

### Bilateral Differences in Shape Factors during Landings from Different Heights

**Figure 4:**
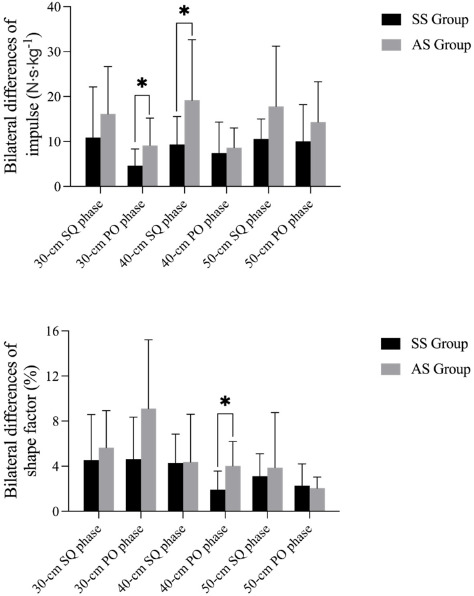
Bilateral differences in the impulse and the shape factor during the landing phase between SS and AS groups. Notes: SQ phase: squat phase; PO phase: push-off phase; * significant differences between the SS and the AS group

Bilateral differences in impulse and shape factors during the landing phase for the SS and AS groups are shown in Figure 4. The results indicate significant bilateral differences in impulses during the squat phase of the landing from the DJ40 (*p* = 0.030) between the SS and AS groups, with no significant difference in the shape factor (*p* = 0.958). The SS and AS groups did not show significant differences in either the impulse or the shape factor at the DJ30 (*p* = 0.255, *p* = 0.473), and the same was observed for the DJ50 (*p* = 0.086, *p* = 0.617).

### Comparison of Bilateral Difference Variables between the Strong and Weak Legs at the DJ

For the DJ30, the time to vGRF was measured; for the DJ40, the vertical loading rate was measured; for the DJ40, the push-off phase shape factor was measured; and for the DJ50, the peak vGRF was measured. No significant Group*Strength interactions were found (*p* > 0.05) (Table 2) nor the main effect for the group.

For the DJ30 push-off phase impulse, the DJ40 squat phase impulse, and DJ30 peak vGRF, no significant Group*Strength interactions were found (*p* > 0.05) nor the main effect for the group, yet there was a main effect for strength (*p* < 0.05, SL > WL).

**Table 2:** Variables with bilateral differences.

					*p* values		
			SS group	AS group	Main effect (Group)	Main effect (Strength)	Interaction (Group ×Strength)
**DJ 30**	time to peak vGRF (ms)	SL	2.04 ± 0.46	1.94 ± 0.61	0.058	0.660	0.417
		WL	2.05 ± 0.46	1.95 ± 0.60			
	push-off phase impulse (N∙s∙ kg^−1^)	SL	1.98 ± 0.21	2.01 ± 0.23	0.939	0.018*	0.409
		WL	1.94 ± 0.18	1.92 ± 0.26			
**DJ 40**	peak vGRF (N∙kg^−1^)	SL	24.35 ± 4.59	25.88 ± 6.61	0.361	0.038*	0.839
		WL	22.34 ± 4.91	24.21 ± 3.83			
	vertical loading rate (N∙kg^−1^/s)	SL	476.55 ± 150.76	491.11 ± 188.95	0.679	0.999	0.503
		WL	482.12 ± 157.61	467.64 ± 101.90			
	squat phase impulse (N∙s∙ kg^−1^)	SL	2.04 ± 0.13	2.06 ± 0.20	0.731	0.001*	0.346
		WL	1.92 ± 0.16	1.85 ± 0.27			
	push-off phase shape factor (%)	SL	70.09 ± 7.26	68.06 ± 7.42	0.725	0.278	0.171
		WL	69.87 ± 7.75	69.88 ± 7.16			
**DJ 50**	peak vGRF (N∙kg^−1^)	SL	27.52 ± 5.30	29.32 ± 8.22	0.686	0.428	0.318
		WL	27.70 ± 6.05	27.75 ± 4.14			

DJ: drop jump; SL: stronger leg; WL: weaker leg; vGRF: vertical ground reaction force; SS: symmetry strength; AS: asymmetry strength; * p < 0.05

## Discussion

Side-to-side limb differences can negatively affect athletic performance (Hart et al., 2014) and increase injury risks on both the DL and the NDL (Edwards et al., 2012; Ford et al., 2003). The difference was calculated by comparing the biomechanical properties of the left and the right leg. Larger discrepancies at ground contact can increase biomechanical disparities and injury risks (Ball et al., 2010; Bates et al., 2013; Paterno et al., 2011; Santello and McDonagh, 1998). Higher DJs produce greater vGRF. Reducing this risk is crucial since high impact during the landing is linked to injuries (Chen et al., 2016; Wang and Peng, 2014). The present study revealed biomechanical differences between the SS and AS groups during jumps; one side of the body experienced an earlier occurrence of peak vGRF or demonstrated a greater force, which could pose a threat of injury

During the DJ, participants aimed to jump as high as possible after the ground contact. Achieving a higher jump relies on both force and time, leading to the concept of impulses (integral of force over time). The shape factor, derived from the impulse and its rectangular area ratio, helps assess performance (Mizuguchi, 2012). Significant differences in impulses were observed between the SS and AS groups in the squat phase at the DJ40 and in the push-off phase at the DJ30. There was also a difference in the shape factor at the DJ40. The AS group exhibited greater differences in the peak vGRF in the squat phase at the DJ40, with a noticeable difference in accumulated impulses. A SL exhibited a greater peak vGRF at the DJ40 than did the WL. Previous studies have shown that the dominant side faces a greater risk of injury due to greater loads (Edwards et al., 2012; Ford et al., 2003).

Previous research has suggested that, compared to those in the DJ20 and the DJ40, no side-to-side differences were observed in variables related to GRF or occurrence time in the DJ60. One study reported that side-to-side differences might occur when the drop height was lower due to shorter ground contact time, which could cause the trailing foot to be unable to land simultaneously with the leading foot (Ball et al., 2010); moreover, previous research has indicated that the ideal drop height for DJs is between 0.40 and 0.62 m (Asmussen and Bonde-Petersen, 1974; Read and Cisar, 2001).

However, if we consider the results related to side-to-side differences in this study, a DJ40 does not appear to be an appropriate height. Additionally, it could be seen from this study that bilateral asymmetry does not always occur; this might be due to an insufficient drop height or too short or long contact time. Even if the subjects were categorized into the AS group, some movements did not show side-to-side differences. Future research should further investigate under which conditions side-to-side differences are likely to occur to avoid or even enhance them.

The present study has some limitations which should be acknowledged. Participants of this study were individuals who regularly engaged in physical fitness classes or training activities; therefore, the results may not be generalizable to those who do not have a habit of exercising or undergoing training. The bilateral leg strength test in this study utilized CMJs as a screening tool to assess asymmetry in leg muscle strength. In the formal research, however, a DJ from a high platform was used as the primary movement for analysis. Additionally, this study was conducted in a laboratory setting where for experimental control, participants were required to stand with their hands on their hips and drop naturally from a high platform. This protocol may differ from real-world scenarios. Whether the participants exerted their maximum effort during each test could be encouraged only verbally, and it was not possible to accurately determine their level of effort.

Future research should include several weeks of training exercises to confirm whether such heights and movements exhibit bilateral differences. Investigating the impact of these discrepancies on sports or competitive events, whether in terms of performance or sports injuries, is worthwhile. This exploration can provide precise and effective guidance for coaches and athletes in training planning.

## Conclusions

In this study, participants underwent CMJ testing and were classified into symmetrical or asymmetrical leg muscle strength groups based on the GRF. The joints were subsequently tested with drop jumps from different heights (30 cm, 40 cm and 50 cm, i.e., DJ30, DJ40 and DJ50, respectively) to examine lower limb biomechanics during the first and second ground contacts and to assess the impact of muscle strength asymmetry on landing injury risk. The results of the study revealed that DJ manoeuvres were predominantly used in training as a means to enhance explosive power. When a DJ40 was set as a training height, multiple variables related to the load during the landing phase exhibited asymmetry compared to drop jumps from heights of 30 cm and 50 cm, resulting in greater impact force values. This finding indicates that the occurrence of a unilateral preference, particularly in activities such as the DJ40, may lead to an elevated risk of lower limb injuries and, if sustained over prolonged periods of training, could amplify the level of asymmetry in the lower limbs.
